# Physician Cross-Cultural Nonverbal Communication Skills, Patient Satisfaction and Health Outcomes in the Physician-Patient Relationship

**DOI:** 10.1155/2012/376907

**Published:** 2012-06-25

**Authors:** Ken Russell Coelho, Chardee Galan

**Affiliations:** Department of Psychology, University of California, Berkeley, CA 94720, USA

## Abstract

Recent empirical findings document the role of nonverbal communication in cross-cultural interactions. As ethnic minority health disparities in the United States continue to persist, physician competence in this area is important. We examine physicians' abilities to decode nonverbal emotions across cultures, our hypothesis being that there is a relationship between physicians' skill in this area and their patients' satisfaction and outcomes. First part tested Caucasian and South Asian physicians' cross-cultural emotional recognition ability. Physicians completed a fully balanced forced multiple-choice test of decoding accuracy judging emotions based on facial expressions and vocal tones. In the second part, patients reported on satisfaction and health outcomes with their physicians using a survey. Scores from the patient survey were correlated with scores from the physician decoding accuracy test. Physicians, regardless of their ethnicity, were more accurate at rating Caucasian faces and vocal tones. South Asian physicians were no better at decoding the facial expressions or vocal tones of South Asian patients, who were also less likely to be satisfied with the quality of care provided by their physicians and to adhere to their physicians' recommendations. Implications include the development of cultural sensitivity training programs in medical schools, continuing medical education and public health programs.

## 1. Background

Patient A has been a patient of physician X for the past 6 months, but he has not had his first physical checkup since he immigrated to the United States from India. Though reluctant, he ultimately complies, yet, with just a single glance at his doctor, he begins to second guess his decision; he has been unexpectedly assigned to a female physician. In some cultures, it is a common cultural practice for patients to be paired up with physicians of their own gender. As the physician begins to ask him a variety of questions, he passively disobeys and responds in single word syllables (i.e., “yes” and “no”). By failing to provide the most accurate information about his medical history, he compromises the quality of his healthcare. The History and Physical (H&P) shows that the patient is in good health, but physician X encourages him to schedule a followup visit. Angry and upset at having been paired with a female physician, he has no intention to ever return for a followup. 

This example demonstrates how a clinician's inability to adequately decode a patient's nonverbal emotional expressions, particularly for a patient of a different cultural background, can contribute to patient dissatisfaction with their physician. Oftentimes, this dissatisfaction is reflected in a patient's failure or refusal to comply and adhere to recommended medical treatments [1]. As the United States patient population increases in ethnic diversity, encounters between physicians and patients from different cultural backgrounds are becoming commonplace [2]. In fact, recent demographic trends suggest that ethnic minorities currently constitute 25 percent of the US population and will be the majority by 2050 (Census Bureau data [3]). Yet, while the general population becomes more diverse, research continues to suggest that the majority of American physicians are overwhelmingly white (Hopkins Tanne, [4]). Simultaneously, research also suggests that a large majority of American physicians fail to appreciate and understand how culture influences their relationships with their patients. Most also lack the skills to effectively bridge potential differences in communication [5]. 

With such challenges, demographic changes, and ethnic disparities, it is becoming increasingly important to understand the factors that affect a patient's satisfaction with a physician of a different cultural background. Of particular interest in the present study is the influence of a physician's ability to decode nonverbal emotions across cultures. 

Although prior research has examined communication between physicians and patients, most of this research has primarily focused on verbal communication, rather than nonverbal [6–8]. However, as argued by Burgoon [9], nonverbal communication in the form of vocal tones and facial expressions enhances verbal communication by serving as the primary medium for the expression of emotions and nonverbal behaviors (Burgoon, [9]). Furthermore, nonverbal behavior is less prone to manipulation because it exists at a more subconscious level (Nardone et al., [10]). Thus, nonverbal communication serves as a better indication of the quality of communication between physicians and patients and is central to identifying strategies for better serving the needs of ethnically diverse patients. Also, evidence already suggests that medical students' nonverbal sensitivity is related to clinically relevant attitudes and behavioral style in a clinical simulation [11]. The literature has also documented an in-group advantage, such that individuals are more accurately able to judge emotions of members of their own cultural group than members of a different one [12, 13]. However, no research currently exists on the impact of cultural differences on patient satisfaction, rapport, and adherence to medical recommendations. The current study addresses these gaps in the existing literature and seeks to enhance our understanding of barriers to building bridges between physicians and patients of different cultural backgrounds. We hypothesize that there is a relationship between a physician's nonverbal skills and a patient's satisfaction and outcomes with his or her physician. Increasing ethnic minority demographics in the United States call for physicians to be selected or trained to be culturally competent. However, this is often not the case, and disparities in minority healthcare continue to persist. Thus, our study seeks to address these disparities by examining a physician's ability to accurately decode nonverbal emotional behaviors across cultures. 

## 2. Methods

### 2.1. Participants

Participants were sampled from physician groups presumed to vary in their exposure to ethnically patient populations. The first part of the study involved 30 physicians, (18 men, 12 women, **M*_age_ = 46.14*, age range: 28–79 years), sixteen of which were of South Asian descent and fourteen of which were of Caucasian American descent, These physicians were recruited from local hospitals, medical centers, community clinics, and educational institutions with clinical practices in the San Francisco Bay Area of Northern California. Only physicians who worked as a practicing medical doctor in a hospital or clinic setting for a minimum of four years and lived in the United States for at least five years were considered to be eligible for the study. Physicians were recruited using email listserves of professional networks and associations. The second part of the study involved survey data obtained from patients recruited from each of the physicians studied in part one. A total of 60 patients (27 men, 33 women, **M*_ age_ = 25*; age range: 19–35 years) were recruited, 30 South Asians and 30 Caucasian Americans. These patients were recruited from clinic lobbies, organizations, and hospitals of the respective physicians during convenient operational hours (12 pm–4 pm, Monday through Friday). Data was collected from a larger number of patients in order to obtain the required sample criteria for the study.

### 2.2. Procedures

The first part of the study, conducted at the respective locations of each physician, examined physicians' abilities to recognize nonverbal cross-cultural emotional expressions. Nonverbal communication was operationalized to include facial expressions and vocal tones, and all stimuli were created and programmed using Media Lab software (2002). After a detailed explanation of the study was provided, consent was obtained from all physicians prior to their participation in the study. All consent forms emphasized the voluntary nature of participation and assured participants that all potentially identifying information would be kept entirely confidential.

After providing consent, each participant was then asked to judge the facial expressions of South Asian and Caucasian American stimuli presented on a computer screen. The present study focused on expressions of anger, fear, disgust, happiness, sadness, surprise, and neutrality. Photographs of Caucasian American faces were drawn from Ekman and Friesen's [14] Facial Affect Coding System (FACS) collection, which has been widely used in previous research. Ekman and Friesen developed their FACS model for prototypical expressions within the United States, which is consistent with American norms for appropriate facial expressions. South Asian faces were drawn from Mandal's collection of South Asian posers (1987). Two separate samples of South Asian Indian judges validated the recognition levels with seventy percent agreement [15]. 

Each facial expression was randomly presented on the computer screen. Since there were two ethnicities of interest and seven different emotional expressions, a total of fourteen different stimuli were presented. After each stimuli was flashed on the computer screen, participants were asked to identify the emotional expression in the picture just presented. They were provided with a list of emotions (i.e., anger, disgust, fear, happiness, sadness, surprise, and neutral expressions) to choose from and could only select one answer. The rest of the study continued until the participants rated all stimuli. All responses were automatically recorded into an excel spreadsheet created by Medialab research software. 

Next, physicians were asked to judge the vocal tones of South Asian and Caucasian American stimuli. Nowicki and Duke's [16] DANVA test, which has been extensively used in previous research and has been well validated, was used for Caucasian American voices. Elfenbein et al.'s [15] validated set of vocal stimuli was used for the South Asian voices. Similar to the previous portion of the study, this part focused on vocal expressions of anger, disgust, fear, happiness, sadness, surprise, and neutral expressions. There were a total of 14 different stimuli presented, and each vocal expression was randomly presented as participants listened using provided headphones. After each stimuli was presented, participants were asked to identify the emotion expressed in the vocal tone by selecting from a list of emotions. The remainder of the study continued as such until all stimuli were presented. The physicians' responses to each vocal tone were automatically recorded into an Excel spreadsheet created by Medialab laboratory research software. 

In the second part of the study, patients completed a self-report survey (see Supplementery Material available online at doi:10.1155/2012/376907) which assessed their satisfaction with their physicians, their adherence to medical treatments, and their desire to continue to see the same physician. Although most of the patients completed the survey immediately upon recruitment (i.e., at the site of their respective clinics, organizations, hospitals, etc.), some, though interested in participating in the study, were in a rush or busy with other tasks. These patients were given the opportunity to complete the survey packet at home and to mail it in at a later time. Detailed consent was obtained from all patients prior to participation in the study.

The first section of the survey (questions 1–4), which measured patient satisfaction, was adapted from Robin DiMatteo's satisfaction scale, which is widely used for communication research in health settings. Extensive research has shown that these questions have high validity and reliability [17–19] (DiMatteo [20]). A sample question assessing patient satisfaction included “On a scale of 1–6, with 1 being very poor and 6 being excellent, rate how well the care you are receiving is meeting your needs.” 

Questions assessing patient adherence and intent to continue care were created for the purposes of the present study. Questions regarding patient adherence included “On a scale of 1–6, with 1 being very poor and 6 being excellent, rate your openness with your physician about your condition/problems.” A sample question assessing patients desire to continue with the same physicians included “If given a choice and the opportunity, would you replace your existing physician with a new physician? Y/N.” 

## 3. Results

Mean accuracy levels for faces and voices were calculated by averaging the scores from each emotion. 

### 3.1. Decoding Accuracy of Facial Expressions

A 2 (ethnicity of physician: Caucasian American or South Asian) × 2 (ethnicity of stimulus: Caucasian American or South Asian) between-subjects analysis of variance (ANOVA) was performed. Physicians' accuracy in identifying the emotions expressed by the facial stimuli was used as the outcome variable. Mean accuracy levels for faces were calculated by averaging the scores from each emotion. This analysis revealed a statistically significant main effect of stimulus ethnicity, *F*(3, 56) = 60.084, *p* < .0001, such that physicians, regardless of their ethnicity, were more accurate at rating Caucasian faces. However, the main effect of physician ethnicity on accuracy scores was statistically insignificant, *p* = .250. The interaction between physician ethnicity and stimulus ethnicity was also insignificant, *p* = .278.

### 3.2. Decoding Accuracy of Vocal Tones

A 2 (ethnicity of physician: Caucasian American or South Asian) × 2 (ethnicity of stimulus: Caucasian American or South Asian) between-subjects analysis of variance (ANOVA) was performed. This time, physician decoding accuracy of vocal tones was used as the outcome variable. This analysis also revealed a statistically significant main effect of stimulus ethnicity (*F*(3, 56) = 35.325, *p* < .0001) such that physicians, regardless of their ethnicity, were more accurate at identifying the emotions expressed in Caucasian voices in comparison to South Asian voices. There was no effect of physician ethnicity on accuracy scores (*p* = .850) and the interaction between physician ethnicity and stimulus ethnicity was also insignificant, *p* = .160. 

### 3.3. Patient Satisfaction

Patients' reports of overall satisfaction with their physicians were subject to a 2 (ethnicity of physician: Caucasian American or South Asian) × 2 (ethnicity of patient: Caucasian American or South Asian) between-subjects ANOVA. Analyses revealed that the main effect of physician ethnicity was statistically insignificant, *p* = .303. However, a main effect of patient ethnicity emerged (*p* = .032) such that Caucasian patients reported being more satisfied with their physicians, regardless of the physician ethnicity. The interaction between physician ethnicity and patient ethnicity was insignificant, *p* = .717. 

### 3.4. Patient Health Outcomes: Adherence to Medical Treatment and Physician Recommendations

A 2 (ethnicity of physician: Caucasian American or South Asian) × 2 (ethnicity of patient: Caucasian American or South Asian) ANOVA was performed. Patient adherence to medical treatment was used as the outcome variable. This analysis revealed a similar pattern of results as the ANVOA performed for patient satisfaction. Although there was an insignificant main effect of physician ethnicity (*p* < .303), the main effect of patient ethnicity was significant (*p* < .001), such that Caucasian patients were more likely to adhere to physician recommendations. There was no significant interaction, *p* = .717. 

## 4. Discussion

The findings of the present study revealed that physicians, regardless of their ethnicity, were more accurate at rating Caucasian faces and vocal tones than South Asian stimuli. Thus, no in-group advantage emerged, and contrary to what might have been expected, in comparison to Caucasian physicians, South Asian physicians were no better at decoding the facial expressions or vocal tones of South Asian patients. The causal reasons for these findings remain unknown. However, it is possible that as individuals matriculate through the advanced learning stages of experience as practitioners in healthcare, their exposure to ethnically diverse populations decreases. Such decreases in exposure to ethnic minorities might be accompanied by a corresponding decrease in an ability to identify the nonverbal emotional expressions of ethnic minorities. These findings suggest that the key to cultivating quality relationships between ethnic minority patients and their physicians does not necessarily rely on increasing the number of minority physicians in the workforce. Instead, training of physicians should incorporate greater exposure to patients of diverse ethnic backgrounds, particularly in environments that are conducive to emotionally didactic and professionally engaging interactions. The vast training and communication programs that have been instituted through hospital education and training and continuing medical education programs must incorporate cross-cultural nonverbal behavioral components into their programs in order to better prepare physicians to meet the needs of the increasingly diverse patient populations. A second possible explanation for these findings is that even though ethnic minorities currently constitute 25 percent of the US population, in comparison to Caucasian individuals, their access to healthcare remains spotty at best. Therefore, the chasm of misunderstanding by Caucasian and South Asian physicians remains deep and wide. Such findings highlight the importance of addressing the disparities in access to healthcare. 

Another notable finding was that South Asian patients were less likely to be satisfied with the quality of care provided by their physicians and to adhere to their physicians' recommendations. Although the causal relationship remains unknown, there is evidence to suggest that some ethnic minority groups evaluate medical care more negatively than white patients [21]. Consistent with the findings discussed above, it is possible that the South Asian patients had higher expectations of healthcare quality and felt less understood and acknowledged by their physicians. 

The current findings must be examined in light of several limitations. First, the study only incorporated static facial expressions and vocal tones. Dynamic channels of nonverbal communication, including proxemics, touch, and hand gestures, are also important factors that affect the quality of relationship between two individuals (e.g., a physician and patient). Second, due to the generally hectic schedules of physicians, there was a particularly small sample size of physicians. Third, although the sample is small, there should have been an analysis of physician gender since this would have been the first study to examine physicians' gender in relation to nonverbal decoding accuracy. Future studies should incorporate larger samples of physicians and include physicians who specialize in different aspects of the medical field. Patients were recruited from the lobbies, organizations, and hospitals of the physicians in our study. These patients could generally be more likely to visit their physicians than those not recruited. Thus, the patients included in the present study might be generally more satisfied with their physicians and more likely to adhere to doctor recommendations than those not recruited. Future studies should incorporate more diverse recruitment strategies. 

Current findings also provide promise for making training design and nonverbal sensitivity for physicians and other allied health care professionals a worthwhile goal given extensive evidence for the day-to-day value of such a skill [22].

## Supplementary Material

Supplementary material include physician and patient consent forms, physician survey, physician and patient recruitment scripts and human subjects research protocol.
