# Supplementary material for: Physician Cross-Cultural Nonverbal Communication Skills, Patient Satisfaction and Health Outcomes in the Physician-Patient Relationship
Source: Int J Family Med. 2012 Jun 25;2012:376907. doi: 10.1155/2012/376907 (PMC3389700; doi:10.1155/2012/376907)
Supplement: Supplementary file 1 — Supplementary material include physician and patient consent forms, physician survey, physician and patient recruitment scripts and human subjects research protocol. [file 376907.f1.docx]

*Appendix A Patient Survey*

**PATIENT SURVEY**

**Privacy**

All information in this survey is anonymous. Please do not mention or write your name anywhere on this survey. The information from

this survey will only be used for the purposes of this study, and will not be disclosed or released for any other purposes wi thout your prior

consent.

**NOTE**: Before you begin this survey, please review the attached consent form, and keep a copy for your record. Physician Code:

Today's Date:

Time: AM/PM

1. Rate each statement by CIRCLING the responses below ranging from strongly agree to strongly disagree

During the examination, this doctor hardly ever tells me what he or she is doing.

• Strongly disagree

• Disagree

• Moderate/Neutral

• Agree

• Strongly agree

This doctor always relieves my worries about my medical condition.

• Strongly disagree

• Disagree

• Moderate/Neutral

• Agree

• Strongly agree

I have some doubts about the ability of this doctor.

• Strongly disagree

• Disagree

• Moderate/Neutral

• Agree

• Strongly agree

My doctor seems to have excellent medical training.

• Strongly disagree

• Disagree

• Moderate/Neutral

• Agree

• Strongly agree

2. Please answer the following questions by CIRCLING your rating from 1-9.

A. How COURTEOUS and RESPECTFUL is your doctor?

1 2 3 4 5 6 7 8 9

Not at all courteous Very Courteous

B. How well does your doctor UNDERSTAND your problem?

1 2 3 4 5 6 7 8 9

Did not understand at all Understood very well

C. How well does your doctor EXPLAIN to you what he/she was doing and why?

1 2 3 4 5 6 7 8 9

Does not explain at all Explains very well

D. Does your doctor USE WORDS that are easy for you to understand?

1 2 3 4 5 6 7 8 9

Uses very hard words Uses very easy words

E. How well does your doctor LISTEN to your concerns and questions?

1 2 3 4 5 6 7 8 9

Did not listen at all Listens very well

F. Does your doctor SPEND ENOUGH TIME with you?

1 2 3 4 5 6 7 8 9

Spent very little time at all Spent as much time as required

G. How much CONFIDENCE do you have in your doctors’ ability or competence?

1 2 3 4 5 6 7 8 9

No confidence Total confidence

H. OVERALL, how satisfied are you with the service that you receive from your doctor?

1 2 3 4 5 6 7 8 9

Completely Dissatisfied Completely Satisfied

**3.** Answer the following questions by CIRCLING one of the following options below each statement.

How likely are you to follow EVERY recommendation from your current doctor?

• Not at all likely

• Not very likely

• Neutral

• Quite a bit likely

• Very likely

How would your rate YOUR overall LIKING for your current doctor?

• Very Poor

• Poor

• Not bad

• Good

• Excellent

How would you describe your overall physical health?

• Very Poor

• Poor

• Not bad

• Good

• Excellent

How completely do you CONFIDE with your doctor in disclosing ALL the details of your medical condition?

• Not at all

• Not very

• Neutral

• Quite a bit

• Very

Given a chance to see a DIFFERENT doctor, how likely would you be to switch?

• Not at all likely

• Not very likely

• Neutral

• Quite a bit likely

• Very likely

How likely are you to RECOMMEND your current doctor to your friends, or other people?

• Not at all likely

• Not very likely

• Neutral

• Quite a bit likely

• Very likely

How likely are you to schedule a FOLLOW UP VISIT with your current doctor?

• Not at all likely

• Not very likely

• Neutral

• Quite a bit likely

• Very likely

How likely would you prefer a doctor of your own ETHNICITY?

• Not at all likely

• Not very likely

• Neutral

• Quite a bit likely

• Very likely

How well do you think that your doctor understands the way you FEEL?

• Not at all well

• Not very well

• Neutral

• Pretty well

• Very well

Where do you usually meet/visit your doctor?

• Hospital

• Clinic/Private office

• Community medical facility

• Educational research facility.

• If other, please SPECIFY

On an average, how much TIME do you spend with your doctor?

• Less than 5 minutes

• 5-10 minutes

• 10-20 minutes

• 20-30 minutes

• 30-40 minutes

• 40+ minutes

4. Fill in the following blank:

How long have you been a patient of your current doctor?

Please mention (Days/months) _
