# Supplementary material for: Physician Cross-Cultural Nonverbal Communication Skills, Patient Satisfaction and Health Outcomes in the Physician-Patient Relationship
Source: Int J Family Med. 2012 Jun 25;2012:376907. doi: 10.1155/2012/376907 (PMC3389700; doi:10.1155/2012/376907)
Supplement: Supplementary file 2 [file 376907.f2.docx]

*APPENDIX B Physician Consent Form*

UNIVERSITY OF CALIFORNIA, BERKELEY

BERKELEY DAVIS IRVINE LOS ANGELES RIVERSIDE SAN DIEGO SAN FRANCISCO SANTA BARBARA SANTA CRUZ

HAAS SCHOOL OF BUSINESS

545 STUDENT SERVICES BUILDING, #1900

BERKELEY, CALIFORNIA 94720-1900

My name is Ken Russell Coelho. I am an undergraduate student researcher in the Psychology Department at the University of California at Berkeley. I am currently working on my honors thesis and would like to invite you to take part in my research study, which looks at the Communication of Emotions.

If you agree to take part in my research, you will be asked to take part in a session of one hour, at the location of your clinic/hospital office and at a time of your convenience. The session will include your participation in a short experiment in the form of a computer task wherein you will view photographs of facial expressions and listen to audio clips of vocal tones, and will be asked to judge the intended emotional state in each of these items. No special training is necessary, your gut judgment of the emotional state in each of these items is all that is requested.

There are no known risks to you from taking part in this research, and no foreseeable direct benefit to you either. However, it is hoped that the research will benefit the scientific community by providing greater understanding of Emotions at the workplace especially in a healthcare setting.

All of the information that I obtain from you during the research will be kept anonymouys. I will store notes about it in a locked file. Each person in this study will have their own code number so that no one other than myself, will know who you are in my notes. The key to the code of names will be kept in a separate locked file and will be destroyed following completion of this study. Your name and other identifying information about you will not be used in any reports of the research.

Your participation in this research is voluntary. You are free to refuse to take part. You may refuse to answer any questions and may stop taking part in the study at any time. Whether or not you choose to take part in this research will have no bearing on your status at your institution.

If you have any questions about the research, you may telephone me, Ken Russell Coelho, at (510) 329 -9386 or by e-mail: [kcoelho@berkeley.edu,](mailto:kcoelho@berkeley.edu) and/or my faculty advisor Prof. Hillary Anger Elfenbein, at (510) 643-9700 or by email: [anger@haas.berkeley.edu.](mailto:anger@haas.berkeley.edu) If you agree to take part in the research, please sign the form below. Please keep the other copy of this agreement for your future reference.

If you have any question regarding your treatment or rights as a participant in this research project, please contact the University of California at Berkeley’s, Committee for Protection of Human Subjects at 510/642-7461, [subjects@uclink.berkeley.edu.](mailto:subjects@uclink.berkeley.edu)

I have read this consent form and I agree to take part in this research

Signature:

Date:
