# Supplementary material for: Physician Cross-Cultural Nonverbal Communication Skills, Patient Satisfaction and Health Outcomes in the Physician-Patient Relationship
Source: Int J Family Med. 2012 Jun 25;2012:376907. doi: 10.1155/2012/376907 (PMC3389700; doi:10.1155/2012/376907)
Supplement: Supplementary file 3 [file 376907.f3.docx]

*APPENDIX C*

*Patient Consent Form*

UNIVERSITY OF CALIFORNIA, BERKELEY

BERKELEY DAVIS IRVINE LOS ANGELES RIVERSIDE SAN DIEGO SAN FRANCISCO SANTA BARBARA SANTA CRUZ

HAAS SCHOOL OF BUSINESS

545 STUDENT SERVICES BUILDING, #1900

BERKELEY, CALIFORNIA 94720-1900

My name is Ken Russell Coelho. I am an undergraduate student researcher in the Psychology Department at the University of California at Berkeley. I am currently working on my honors thesis and would like to invite you to take part in my research study, which looks at the communication of emotions.

If you agree to take part in my research, you will be asked to fill out a short paper survey of 10 minute s in which you will be asked questions about the relationship that you as a patient, have with your physician. If you have time, you may complete the survey now in the waiting room of the clinic/hospital of your family physician and hand it back to me. Please do not write your name or any other personal identifying characteristics on it. If you do not have time to complete the survey right now, you may mail it to me anonymously at a later time. A self addressed stamped envelope is provided to you for that purpose.

There are no known risks to you from taking part in this research, and no foreseeable direct benefit to you either. However, it is hoped that the research will benefit the scientific community by providing greater understanding of doctor-patient communication in a healthcare setting. You will not sign the consent form or write down your name anywhere and as a result cannot be identified.

All of the information that I obtain from you during the research will be kept anonymous. I will store notes about it in a locked file. There will be no identifying characteristics/names on any of the forms and responses cannot be identified and will not be shared with your physicians. There will be no direct identifiers on the survey or any of the forms for this study. However, there will only be a physician code to ensure participation of an adequate amount of patients for each physician studied. No other information about you will be used in any reports of the research.

Your participation in this research is voluntary. You are free to refuse to take part. You may refuse to answer any questions and may stop taking part in the study at any time.

If you have any questions about the research, you may telephone me, Ken Russell Coelho, at (510) 329 -9386 or by e- mail: [kcoelho@berkeley.edu,](mailto:kcoelho@berkeley.edu) and/or my faculty advisor Prof. Hillary Anger Elfenbein, at (510)643-9700 or by email at: [anger@haas.berkeley.edu.](mailto:anger@haas.berkeley.edu) If you agree to take part in the research, please read this form and verbally acknowledge your consent to participate. Please keep a copy of this agreement for your future reference.

If you have any question regarding your treatment or rights as a participant in this research proje ct, please contact the University of California at Berkeley’s, Committee for Protection of Human Subjects at 510/642 -7461, [subjects@uclink.berkeley.edu.](mailto:subjects@uclink.berkeley.edu)
