# Supplementary material for: Physician Cross-Cultural Nonverbal Communication Skills, Patient Satisfaction and Health Outcomes in the Physician-Patient Relationship
Source: Int J Family Med. 2012 Jun 25;2012:376907. doi: 10.1155/2012/376907 (PMC3389700; doi:10.1155/2012/376907)
Supplement: Supplementary file 4 [file 376907.f4.docx]

*APPENDIX D Patient Recruitment Script*

My name is Ken Russell Coelho. I am an undergraduate student researcher in the Psychology Department at the University of California at Berkeley. I am currently working on my honors thesis and would like to invite you to take part in my research study, which looks at the communication of emotions.

If you agree to take part in my research, you will be asked to take part in a 10 minute short paper survey in this waiting room of the clinic/hospital of your family physician. You will basically be asked questions about the relationship that you as a patient, have with your physician.

Your participation in this research is voluntary. You are free to refuse to take part. You may refuse to answer any questions and may stop taking part in the study at any time.

There are no other known risks to you from taking part in this research, and no foreseeable direct benefit to you either. However, it is hoped that the research will benefit the scientific community by providing greater understanding of doctor-patient communication in a healthcare setting.

Do you have any questions for me? (I will address any query or concern the patient brings up to make him/her feel comfortable)

If you would like to take part in this research study, please look over the following consent form and please keep one copy of the agreement for your future reference. Once again, do you have any further questions for me?

Here is the survey (containing both the screening form and questionnaire in one packet).

If you have time, you may complete the forms now and hand it back to me. Please do not write your name or any other personal identifying characteristics on it.

If you do not have time to complete the survey right now, you may mail it to me anonymously at a later time. Here is a self addressed stamped envelope that you can use to mail it to me.

Since this study has received approval from UC Berkeley, if you have any question regarding your treatment or rights as a participant in this research project, you may contact the University of California at Berkeley’s, Committee for Protection of Human Subjects. Their contact information can be found on your copy of the consent forms that you have just signed.

Thank you for your time and have a great day!
