# Supplementary material for: Physician Cross-Cultural Nonverbal Communication Skills, Patient Satisfaction and Health Outcomes in the Physician-Patient Relationship
Source: Int J Family Med. 2012 Jun 25;2012:376907. doi: 10.1155/2012/376907 (PMC3389700; doi:10.1155/2012/376907)
Supplement: Supplementary file 5 [file 376907.f5.docx]

*APPENDIX E*

*Committee for the Protection of Human Subjects Protocol Document*

**Investigator: Ken Russell Coelho**

**Advisor: Hillary Anger Elfenbein**

**#1 Title**:-Effects of the ability to accurately recognize and perceive nonverbal communication of emotions across cultures: influence on satisfaction and outcomes in the ‘Physician-patient relationship’

**#2 Related Projects**:-CPHS #2003-9-28, Title: Testing Expressive Style and Exposure as a

Mechanism of Accuracy in Emotion Recognition

**#3 Purpose:-**How does cross-cultural communication affect the doctor patient relationship? Much research has focused on verbal communication skills of physicians and its influence on patient satisfaction, compliance and enhancing the Physician-patient relationship which is highly recognized. However, while nonverbal communication has been shown to be essential to patient care, and the ability to read emotion has shown a positive impact on rapport between a physician and patient, the role and influence of cross-cultural differences on doctor patient nonverbal communication has not been addressed and has never been tested. Recent empirical findings document cross-cultural differences in the recognition of nonverbal emotions. With minorities increasing in number and physicians not being selected or trained to be culturally competent, disparities in minority healthcare need to be addressed. The proposed study seeks to address these disparities, by studying and testing the influence of a physician’s ability to accurately decode nonverbal emotional behaviors across cultures on satisfaction and outcomes in doctor patient interactions. A

2 part study will directly examine the Physician-patient relationship through nonverbal facial expressions and vocal tones as emotive cross-cultural stimuli and patient self reports which will provide insight on

patients’ satisfaction and outcomes.

**#4 Subjects:** -Part 1 of the study will involve 50 physician subjects from Eden Medical Center, Castro Valley, CA, Washington Hospital, Fremont, CA, Kaiser Permanente, Hayward, CA, Children’s Hospital, Oakland, CA & Stanford University Medical Center, Palo Alto, CA. Part 2 of the study will involve at least

200 patient subjects (2 patients of each physician studied), sampled from the patient pool of each physician in order to include at least two Caucasian and two Indian patients for each doctor.

**#5 Recruitment:-**I am currently speaking with managers and administrative personnel at the above mentioned Hospitals identified via professional contacts. I will go over the entire logistics of the study, from physician and patient recruitment, to what will be required for participation in the study with the administrative personnel and managers of the hospitals. A copy of the UC Berkeley CPHS approval letter will also be provided to them to include in their records. The Administrative personnel and managers will provide physician rosters and information in order to recruit the required participants in the study. Although none of them have confirmed their participation, one has already expressed interest and willingness to consider taking part in the project. Documenting that the current proposal receives CPHS approval will enhance the hospital’s willingness to take part in the study. Once we have permission to approach specific

physicians, we will send a written invitation to each individual randomly, which is attached below. This invitation emphasizes the voluntary nature of taking part in the study.

For the first part of this study, physician subjects will be randomly selected from the hospital rosters which will be obtained from the hospital administrative personnel, half of which will be Caucasian American and the other half will be Indian (India). Physician subjects will be identified and chosen ideally in June- July’04. The Caucasian and Indian physicians will be selected from the printed rosters based on the apparent ethnicity of their names, and care will be taken to include only those physicians that have names that sound completely Indian and Caucasian. Any inconspicuous sounding names or names that do not represent either ethnic origin will not be invited to participate in the study. The gender of the physician will also be established from their names. The pool will represent a random sample from the total eligible in this manner, and it is hoped that it will include equal proportions of similar age groups. In the event the pool does not represent equal proportions of age groups, the same will be mentioned in the research article. No special screening measure will be used to include an equal proportion of age groups. When choosing and recruiting physicians for the study, I will be aided by the Recreation therapist of the Eden Medical Center, who will send out memos of my study to the selected physicians. I am working with Staff of the other hospitals to determine physician subjects in order to increase diversity of subject pool.

For the second part of this study, the patient pool will be recruited during usual non busy times (12 noon-

4pm) of the day in the waiting rooms of the hospital/ physician’s clinic. The pool will represent equal proportions of both gender and similar age groups. This part of the study will be completed concurrently while running physician subjects in Part 1. Because it is not possible to screen patient names in advance, and because their participation is relatively brief, a range of patients will be approached. Any information collected from individuals who are not Caucasian or Indian, and thus who do not meet the criteria of the study, will be destroyed.

**#6 Screening Procedures:-**Random Selection of physician and patient subjects based on ethnic background in order to maintain the cross-cultural balanced environment necessary for a cross-cultural study of such a nature**.** As mentioned above, for the physicians, the physicians name will be used to screen for his/her ethnic background and gender. In the event the physicians name and ethnic background do not match, the physician subject will still be run as a subject in this study, however, his/her information will be destroyed and will not be used for the purposes of this study**.** No particular screening measure will be used to include similar age groups in the participant pool of the physicians. No other particular screening procedures will be used for the physicians.

For the patients, 4 patients of each physician included under Part 1 will be asked to complete a 1 page pre**-** screening survey in which they will be asked biographical information about themselves (their age, gender, and ethnic background). The patient will be free to disregard any question he/she feels uncomfortable answering. Please find attached, a copy of this 1 page prescreening survey. This survey will help provide information on the patients’ age, gender and ethnic background for this study. This information will only be used for screening purposes and once eligibility is determined, all information obtained from these surveys will be destroyed. No other particular screening procedure will be used for the patients.

**#7 Data Collection Procedures:**-

*Part 1 (June – December’04): Effects of cross-cultural non verbal emotions on Physicians’ decoding accuracy*

This part of the study will examine the influence of cross-cultural nonverbal emotions on decoding accuracy. This part of the experiment will take place at the location of the particular physicians’ hospital office/clinic at a time of his/her convenience. The session will last for duration of 1 hour. The session will include participation in a short experiment in the form of a computer program (Medialab, 2002) task wherein

the physician will view photographs of facial expressions and listen to audio clips of vocal tones, and will then be asked to judge the intended emotional state in each of these items. No special training is necessary, only gut judgment of the emotional state in each of these items is all that is requested from the physician subjects. The results of this computer task will automatically be recorded in a data file that gets created after every subject completes the task. Immediately after the session, the physician subject will be requested to ask any questions he/she may have about the study. No special debriefing will be required because of the open nature of this study. This study does not involve deception of any kind, and there will be no need for any follow up sessions with the physician subjects.

This study will employ a fully balanced multiple choice judgment design that has been proven to be reliable and valid by Hillary Anger Elfenbein et al (2002). An experiment will be created and programmed with Media Lab (2002) Laboratory software using a standard emotion recognition judgment task that involves viewing photographs of facial expressions and listening to vocal tones. This same emotion recognition measure has been used in other studies without incident, for example in the project approved under CPHS #2003-9-28. The measure will be administered in July-December’04.

The photographs of facial expressions will be from 2 distinct cultural groups namely Caucasian American and Indian (India). Ekman and Friesen’s (1976) Facial Affect collection pictures using the Facial Affect Coding System (FACES; Ekman & Friesen, 1978) will be used as the American cultural stimuli primarily because it is consistent with American norms for appropriate facial expressions and also due to its wide popularity in emotion recognition research. Pictures of facial expressions of Indian posers, posing to an imagined emotional situation will be used as the Indian (India) cultural stimuli. This set of photographs was created by an associate of my mentor (Mandal, 1987), and has been used in previous research (e.g., Elfenbein et al., 2003). The following facial expression emotions will be used: anger, disgust, fear, happiness, sadness, surprise and neutral. All participants will view emotional expressions posed by each cultural group. This arrangement will help create a cross-cultural environment in order to study nonverbal communication. Each photograph will appear randomly on the laptop screen for every participant. Exposure to each picture will be followed by a few survey questions that will be used in order to elicit decoding of specific emotions, viz. ‘How does the person in the picture feel? ‘On a scale of 1-5 with 1 being not certain at all and 5 being absolutely certain, how sure are you?’ The answers to each picture and reaction time will be recorded and analyzed against the standards that have been set for each photograph set.

Likewise, there will be a judgment task of the ability to understand vocal expressions of emotion. The Diagnostic Analysis of Nonverbal Accuracy (DANVA) developed at Emory by Steve Nowicki

[Nowicki, S., Jr., & Duke, M. P. (1994) test, which is widely used and highly validated, will be used for

the American voices. For the Indian voices, Mandal and Elfenbein’s new set of vocal stimuli, which are currently undergoing validation (under the auspices of project CPHS #2003-9-28), will be used. The

physician participants will listen to audio clips of vocal tones and record a multiple-choice response as

to the emotion intended in the vocal tone.

The entire first part of the study will help measure the accuracy of physicians’ ability to recognize

nonverbal behavior in cross-cultural communications

*Part 2 (August - December’04): Patient satisfaction and Outcomes in the Physician-patient relationship*

The second part of this study will analyze patient satisfaction and outcomes in the Physician-patient relationship. Indian and Caucasian American patients will be studied from the Caucasian physicians’ patient pool and vice versa for the Indian physician patient pool in order to maintain the cross-cultural environment.

The mentioned screening procedure (as mentioned in #6) will be used to recruit patients. At least four patients of each physician studied in part 1 will be asked to complete a standard survey questionnaire (after being administered the 1 page pre-screening survey) in order to assess patient satisfaction, effective Physician-patient relationship and ultimate outcomes. This survey will be administered in the form of a paper survey packet in the waiting room of the office/clinic of the physician. The patient will not be asked to write his/her name on the survey and there will be no way in which individual patient responses will be linked to themselves individually. Patient responses will also not be linked to their physician’s responses in any way. Questions will be asked ranging from relationship duration, physicians’ ability to assess medical problems to patients comfort and effective communication. The questions will be selected from the following list of potential survey items:

1. ‘How long have you been a patient of your current Physician? (fill in)

2. How long have you known your current Physician? (fill in)

3. In the past 2 months, how many times have you seen your current physician for an appointment? (fill in)

4. On an average, how many times do you see your current physician in a year? (fill in)

5. PLEASE CIRCLE: On an average, how much of time do you spend with your physician during a regularly scheduled appointment?

a) 5-10 min

b) 10-20 min c) 30-40 min d) 50+min

6. PLEASE CIRCE: Would you rate the quality of healthcare from your physician as EXCELLENT? Y/N

7. On a scale of 1-6 (1=very poor, 2=poor, 3=fair, 4= good, 5=very good, 6=excellent), rate your

Physicians’…

a) Quality of examinations

b) Active listening to your needs and condition c) Answers to your questions

e) Friendliness and courteousness shown to you

f) Eye contact with you during regular appointments g) Body language shown to you during appointments h) Emotional expressiveness shown to you

i) Physical/Tactile contact with you

j) Communication between himself/herself and his/her office/medical/hospital staff k) Communication between him/her and yourself

l) Reassurance and support offered to you

m) Advice about ways you could avoid illness and stay healthy n) Receptiveness to speaking with you over the phone

o) Concern/empathy for your medical condition

p) Concern/empathy for your overall social/emotional/mental condition

8) On a scale of 1-6 (1=very poor, 2=poor, 3=fair, 4= good, 5=very good, 6=excellent), rate…

a) Your ease of overall speaking to your physician

b) Your ease of getting referrals to specialized medical treatments or other specialist physicians c) The comfort of the hospital/clinic environment during appointments

d) Your openness with your physician about your condition/problems e) Your trust in your physician

f) How well the care you are receiving is meeting your needs

9. PLEASE CIRCLE:

a) Your usual mood during a regular appointment visit: Happy, sad, anger, disgust, fearful, frustration, overwhelmed

b) Currently, your Health is

EXCELLENT, VERY GOOD, GOOD, FAIR, POOR

c) Overall, Are you satisfied with your Physician? Y/N

d) Could the healthcare you are receiving be improved? Y/N

e) If yes, what specifically could be improved (fill in)

e) If given a choice and the opportunity, would you replace your existing physician with a new physician? Y/N

Finally, patients will be asked to identify their family’s cultural background using the following questions:

10. What is your family’s country of origin? (fill in)

11. What is your country of birth? (fill in)

11. What was your mother’s country of birth? (fill in)

12. What was your father’s country of birth? (fill in)

This second part of the study will assess patient satisfaction in order to shed light upon whether the patient has a good working relationship with his/her physician. The final questions will allow us to analyze separately patient satisfaction from members of different cultural groups as a function of the doctor’s relative familiarity with emotional expressions from that cultural group.

References:

Carrillo, J., E., Green, A., R., & Betancourt, J., R. (1999) *Cross-cultural primary care: A patient-based approach. Annals of Internal Medicine. v130, 10, 829-834*

DiMatteo, M. R., Friedman, H. S., & Taranta, A. (1979). Sensitivity to bodily nonverbal communication as a factor in practitioner-patient rapport. *Journal of Nonverbal Behavior, 4, 18-26.*

DiMatteo, M. R., Hays, R. D., & Prince, L. M. (1986). Relationship of physician's nonverbal communication skill to patient satisfaction, appointment noncompliance, and physician workload. Health Psychology, 5, 581-594.

DiMatteo, M. R., Taranta, A., Friedman, H. S., & Prince, L. M. (1980). *Predicting patient satisfaction*

*from physician’s nonverbal communication skills. Medical Care, 18, 376-387.*

Ekman, P. (1972). Universals and cultural differences in facial expressions of emotion. In J. Cole (Ed.),

*Nebraska Symposium on Motivation, 1971* (Vol. 19, pp. 207-282). Lincoln: University of Nebraska Press

Elfenbein, H. A., & Ambady, N. (2000). A meta-analysis of the universality and cultural specificity of emotion recognition. *Paper presented at the 12th Annual Meeting of the American Psychological Society, Miami, Florida*.

Elfenbein, H. A., & Ambady, N. (2002a). On the universality and cultural specificity of emotion recognition: A meta-analysis. *Psychological Bulletin, 128,* 203-235.

Elfenbein, H. A., & Ambady, N. (2002a). Is there an in-group advantage in emotion? *Psychological*

*Bulletin, 128*, 243-249.

Ford, S., Fallowfield, L., & Lewis, S. (1996). *Doctor-Patient Interactions in Oncology. Social Science in Medicine, 42, 11, 1511-1519*

Gillis, J. S., Bernieri, F. J., & Wooten, E. (1995). The effects of stimulus medium and feedback on the judgment of rapport. *Organizational Behavior and Human Decision Processes*, 63, 33-45.

Griffith, C., H., Wilson, J., F., Langer, S., & Haist, S., A. House Staff nonverbal communication skills and standardized patient satisfaction. *Journal of General Internal Medicine, 18, 170-174*

Lee, S., J., Back, A., L., Block, S., D., & Stewart, S., K. (2002) Enhancing Physician Patient

Communication. *Journal of the American Society of Hematology. 464-483*

Nowicki, S., Jr., & Duke, M. P. (1994). Individual differences in the nonverbal communication of affect: The diagnostic analysis of nonverbal accuracy scale. *Journal of Nonverbal Behavior, 19,* 9-35.

Singer, M., K., & Lakha, S. K. (2003). A Strategy to Reduce Cross-cultural Miscommunication and

Increase the Likelihood of Improving Health Outcomes. *Academic Medicine*, v78, 6, 577-587

Tervalon, M., (2003) Components of Culture in Health for Medical Students' Education. *Academic*

*Medicine*, v78, 6, 570-576

**#8 Benefits: -** There are no direct benefits to subjects. For the physician subjects, completing the facial expression and vocal tone recognition task will provide individual feedback on one’s own ability to understand nonverbal communication better.

**#9 Risks:** - There are no serious risks to the participants in this study. Although there exists risks of embarrassment in the event patient responses were revealed inadvertently, there is no way their responses can be directly liked to their names since each participant will be given a unique code. All the information that is collected for the purposes of this study, will be kept strictly anonymous in a locked file. Other risks are no greater than would typically be encountered in a Hospital setting. Participation will be voluntary and anonymous and therefore in no way allowed to be used as a Hospital to physician employee appraisal tool. The use of measures to ensure strict anonymity limits any other risk to the individuals.

**#10 Anonymous Measures: -** Participants will be informed via the consent form that all of the information obtained via the research will be kept anonymous. The data from each participant will be given a unique code so that names cannot be directly linked to the data. The key to the code of names will be kept in a separate locked file from the test data, to be used for the generation of individualized feedback reports, and will be destroyed following the completion of the study.

**#11 Informed consent:-**Participants will receive a document providing details about the nature of the study, in order to ensure their informed consent. Attached to this application is a copy of the consent form that will be used. Before the start of the experiment, the subjects will receive a *consent form* containing an invitation to participate in this study and will be asked to acknowledge receipt and sign it on agreeing to participate in this study.

**#12 Financial Aspects:-**Participants will not be paid for taking part in this study.

**#13 Written Materials:-**The FACES and DANVA test that are included in the current study are from the published articles listed above. Other than the above mentioned written materials and patient satisfaction questions, no new written materials will be generated for the purpose of this study.
